# Increasing number of cervical examinations in term labor does not increase the risk of maternal intrapartum fever

**DOI:** 10.1002/pmf2.70036

**Published:** 2025-05-23

**Authors:** Chase Calvert, Miriam Alvarez, Jeny Ghartey, Elaine Avshman, Sama Jwaied, Nandini Raghuraman, Lorie M. Harper, Alison G. Cahill

**Affiliations:** ^1^ Department of Women's Health Dell Medical School University of Texas at Austin Austin Texas USA; ^2^ Texas A&M School of Medicine Bryan Texas USA; ^3^ Department of Obstetrics & Gynecology Washington University School of Medicine St. Louis Missouri USA

**Keywords:** cervical exam, clinical chorioamnionitis, intrapartum fever, labor, labor management, maternal fever, term pregnancy

## Abstract

**Introduction:**

Intrapartum fever, a significant cause of maternal and neonatal morbidity, often arises from ascending infections into the uterus. Digital cervical examinations may facilitate the introduction of microorganisms, theoretically increasing fever risk. Prior studies on the association between exam frequency and fever have yielded inconsistent findings, a discrepancy that may be influenced by the choice of analytic method. We aimed to evaluate the dynamic relationship between cervical exam frequency and maternal fever risk using time‐to‐event analysis, modeling risk of fever as it evolves over the duration of labor.

**Methods:**

This retrospective cohort study included 3627 singleton pregnancies delivering at ≥37 weeks over a 6‐year period. Patients were included if they reached the second stage of labor. Patients were categorized into three groups of number of cervical exams based on their natural distribution within the cohort to reflect clinical practice. Time‐to‐event analyses with Cox proportional hazard models were used to account for labor duration, adjusting for induction, Bishop score > 5, rupture of membranes (ROM), and interaction of ROM with time. A secondary analysis was performed by redefining time zero as the time of membrane rupture. Subgroup analyses included nulliparas and those admitted with less than 6 cm cervical dilation.

**Results:**

Among the 3627 patients, 160 (4.1%) developed intrapartum fever. In the unadjusted analysis, the risk of fever was higher in patients with more cervical exams: 1.5% in the 1–4 exam group, 4.1% in the 5–6 exam group, and 9.6% in the ≥7 exam group. However, after adjusting for confounding factors using time‐to‐event analysis, no increased risk of fever was observed with more exams. In fact, patients with ≥7 exams had the lowest risk (HR 0.24, 95% CI 0.13–0.43) compared to those with 1–4 exams. A similar association was demonstrated in subgroup analyses of nulliparous patients (*p* < 0.001) and those admitted with less than 6 cm cervical dilation.

**Conclusion:**

In this time‐to‐event analysis, no association was found between an increased number of cervical examinations during labor and an elevated risk of maternal fever. The observed increased risk of intrapartum fever in prior studies using logistic regression may be influenced by confounding factors such as labor duration, rather than a direct causal relationship. These findings suggest that cervical exams, when clinically indicated, do not inherently increase the risk of fever.

## 1 INTRODUCTION

Fever in labor is a significant contributor to maternal and neonatal morbidity. The etiology of intrapartum fever is multifactorial but is mostly attributed to intraamniotic infection caused by the ascent of pathogens from the lower genital tract into the uterine cavity [1]. Digital cervical examinations, a routine component of labor management, have been identified as a potential risk factor for infection by facilitating the introduction of microorganisms into the uterine environment [2, 3, 4]. Despite growing efforts to standardize labor management protocols to improve outcomes and reduce disparities, uncertainty remains about how cervical exam frequency influences fever risk, underscoring the need for clearer evidence to guide the appropriate timing and frequency of these exams within labor management guidelines [5, 6, 7].

Several retrospective studies have examined the relationship between cervical exam frequency and intrapartum fever, but the findings have been inconsistent, largely due to differences in analytical approaches. Retrospective studies that have used logistic regression have frequently reported an increased risk of fever with more cervical exams [8, 9, 10, 11]. However, logistic regression models often treat labor duration and exam frequency as independent variables, despite their inherent collinearity. Longer labors naturally lead to more frequent cervical exams, and logistic regression may struggle to separate these intertwined variables, potentially leading to an overestimation of the independent effect of cervical exams on fever risk. This limitation may help explain the conflicting results across studies.

In contrast, a prior single‐center study using time‐to‐event analysis found no link between cervical exam frequency and fever risk [12]. By modeling risk over time, such an analytic approach accounts for changes in factors such as labor duration and membrane rupture without treating them as independent variables. Considering the contrast between these findings and those from other studies using different analytic techniques, we sought to replicate a time‐to‐event analysis in a more contemporary cohort, with varied patient characteristics and labor management practices, to assess the reproducibility of the results.

The objective of our study was to evaluate the relationship between cervical exam frequency and intrapartum fever risk in a contemporary cohort of singleton pregnancies at a large, quaternary care hospital, that houses both academic and community obstetric practices. Using time‐to‐event analysis, we modeled fever risk as it evolves over the course of labor, incorporating dynamic factors such as labor duration and the timing of membrane rupture as time‐dependent variables.

## 2 MATERIALS AND METHODS

This retrospective cohort study included 3627 patients with singleton pregnancies delivering at or beyond 37 weeks’ gestation between 2016 and 2022 at a quaternary care hospital in the South. Our hospital has a diverse labor management environment with both academic and community practices. This includes university practices with standardized labor induction and labor management protocols, with cervical checks performed by residents and labor managed by residents and fellows with attending supervision. The labor floor also houses non‐academic community/private practices, wherein non‐university physicians and certified nurse midwives manage labor, and nurses perform cervical checks.

Patients were included if they had a singleton, vertex pregnancy at term and progressed to the second stage of labor. Exclusion criteria included cesarean delivery before the second stage, documented fever on admission, known major fetal anomalies, multiple gestation, or intrauterine fetal demise. Patients with a history of prior cesarean delivery were excluded to ensure a more homogenous cohort and to control for potential confounders, as TOLAC patients may experience different management practices—such as in monitoring, labor augmentation, and decision‐making—that could affect labor outcomes and complicate the analysis of cervical exam frequency and fever risk.

The primary outcome was maternal fever, defined as a temperature ≥38.0°C during the first or second stage of labor. The primary exposure was the number of digital cervical examinations during the first stage only. We could not accurately account for or reliably assess the number of exams during the second stage and while pushing.

Labor inductions are typically initiated within an hour of arrival, and cervical exams are performed within 30 min of rooming. Per institutional protocol, cervical checks are performed every 4 h in latent labor (0–6 cm dilation) and every 2 h in active labor (6 cm to complete dilation) [13]. In the academic setting, where residents are continuously available, cervical exams are generally timelier and more consistent. However, with community providers, there may be greater variability in the frequency of exams based on individual practices and provider availability. Data were collected from the electronic medical record, including demographics, time‐stamped cervical exams, maternal vital signs, the length of each stage of labor, labor interventions, and mode of delivery.

Patients were grouped based on the frequency of cervical exams and compared with respect to baseline and labor characteristics. Exam categories were defined based on their natural distribution within the cohort to reflect clinical practice and avoid arbitrary cutoffs. The 1–4 exam group was selected as the reference group as it represents expected exam frequency during spontaneous, uncomplicated labors. The second group had 5–6 exams, and the third group had 7 or more exams. Time zero was defined as the time of the first cervical exam, and total exposure time was measured from the first exam through either the development of fever or delivery. This included time spent in both the first and second stages of labor. Patients were censored at delivery if they did not develop fever.

Statistical analysis included the χ^2^ test or Fisher's exact test for dichotomous outcomes. Continuous variables were assessed for normality using the Shapiro–Wilk test; normally distributed variables were compared using the Student's *t*‐test; and non‐normally distributed variables were compared using the Mann–Whitney U test. Less than 1% of fields were missing for any variable, so imputed values were not used.

A Cox proportional hazards model was used to evaluate the relationship between the number of cervical exams and the risk of fever, incorporating the time‐dependent nature of labor. We hypothesized that the effect of rupture of membranes on fever risk may vary over time in labor. To investigate this, we included an interaction term between the rupture of membranes and time in our Cox proportional hazards model, using maximum likelihood estimation. The significance of this interaction was assessed with the Wald test, and hazard ratios were calculated at different labor stages to interpret the interaction, ensuring its significance and consistency.

The model was adjusted for potentially confounding factors identified in the stratified analyses and those that have been historically described to be associated with the risk of maternal intrapartum fever. The models were ultimately adjusted for induction of labor, Bishop score > 5 on admission, rupture of membranes on admission, and interaction of rupture of membranes with time. Non‐significant factors were removed stepwise. The proportional hazards assumption was tested using the Grambsch‐Therneau method [14]. The model was rerun with an additional adjustment for epidural anesthesia to account for any fever potentially associated with its use.

To examine the effect of cervical examination frequency on fever risk after membrane rupture, we conducted a secondary analysis with time zero defined as membrane rupture. We also conducted a priori subgroup analyses on (1) nulliparous patients, whose labor courses differ significantly from multiparas, and (2) patients admitted in latent labor (< 6 cm dilation), including those undergoing induction. The latter group, consisting of 89.3% of the cohort, includes patients in the latent phase of labor, who typically experience a longer duration of labor and more frequent cervical exams. These analyses were designed to account for confounding by parity and labor duration, both of which influence cervical exam frequency and fever risk.

The required sample size was 3514 observations, assuming a 7% incidence of fever, *R* ^2^ of 0.4, alpha of 0.05, and 80% power. Statistical analyses were performed using SAS 9.4. This retrospective study utilized de‐identified medical records, and as such, informed consent was waived by the Institutional Review Board due to the minimal risk involved and the inability to directly contact patients. All patient data was analyzed in accordance with HIPAA regulations, ensuring strict confidentiality.

## 3 RESULTS

Among the 3627 patients meeting inclusion criteria, 160 (4.1%) developed an intrapartum fever. Table 1 details the baseline characteristics of the cohort, grouped by number of exams. Patients with a greater number of cervical examinations (7 or greater compared to reference) had some notable statistically significant differences: they were more likely to be admitted with a lower Bishop score (median 3 compared to 5) and lesser cervical dilation (1 cm compared to 4 cm). They were less likely to undergo spontaneous vaginal delivery. They were more likely to require cervical dilation with prostaglandins and more likely to be nulliparous.

**TABLE 1:** Characteristics of analytic sample, grouped by number of cervical exams.

	Number of cervical exams			
	1–4 (*n* = 1473)	5–6 (*n* = 1233)	More than 7 (*n* = 921)	*p* value
Patient's age, median (IQR)	30 (25, 34)	30 (25, 34)	30 (25, 34)	0.1513
Intrapartum fever				
No fever	1451 (98.5)	1183 (95.9)	833 (90.4)	< 0.0001
Fever	22 (1.5)	50 (4.1)	88 (9.6)	
Parity				
Multiparous	996 (67.6)	625 (50.7)	320 (34.7)	< 0.0001
Nulliparous	477 (32.4)	608 (49.3)	601 (65.3)	
Gestational age, median (IQR)	39 (38, 39)	39 (38, 39)	39 (38, 40)	0.3001
Prostaglandin				
No	1261 (85.6)	836 (67.8)	420 (45.6)	< 0.0001
Yes	212 (14.4)	397 (32.2)	501 (54.4)	
Mode of delivery				
Cesarean	29 (2.0)	66 (5.4)	98 (10.6)	< 0.0001
Operative vaginal delivery	61 (4.1)	71 (5.8)	73 (7.9)	
Vaginal	1383 (93.9)	1096 (88.9)	750 (81.4)	
Admission Bishop score, median (IQR)	5 (3,7)	4 (2, 5)	3 (1, 4)	< 0.0001
Cervical dilation on admission, median (IQR)	4 (2, 5)	2 (1, 4)	1 (1, 3)	< 0.0001
Labor type				
Induction	365 (24.8)	451 (36.6)	420 (45.6)	< 0.0001
Spontaneous	730 (49.6)	342 (27.7)	152 (16.5)	
Augmentation	376 (25.5)	437 (35.4)	344 (37.4)	
Bishop score on admission				
Bishop score ≤ 5	754 (51.2)	941 (76.3)	807 (87.6)	< 0.0001
Bishop score > 5	719 (48.8)	292 (23.7)	114 (12.4)	
Rupture of membranes on admission				
No ROM on admission	1137 (77.2)	1057 (85.7)	838 (91.0)	< 0.0001
ROM on admission	319 (21.7)	174 (14.1)	80 (8.7)	
Unknown/NA	17 (1.2)	2 (0.2)	3 (0.3)	

*Note*: Data are *n* (%) unless otherwise specified.

Abbreviations: IQR, interquartile range; SD, standard deviation.

To provide a clearer understanding of the relationship between cervical exam frequency and intrapartum fever risk, we compared the results from two different analytic approaches. In the unadjusted analysis presented in Table 2, we observed a significant increase in the odds of fever with a higher number of cervical exams. Specifically, compared to patients with 1–4 exams, those with 5–6 exams had nearly three times the odds of developing fever (OR 2.79, *p* < 0.0001), while those with 7 or more exams had nearly seven times the odds (OR 6.97, *p* < 0.0001).

**TABLE 2:** Unadjusted fever incidence and odds ratios for fever by cervical exam group.

Number of cervical exams	Fever incidence (%)	Odds ratio (OR)	95% Confidence interval (CI)	*p* value
1–4 (*n* = 1473)	1.5	1.00	reference	—
5–6 (*n* = 1233)	4.1	2.79	(2.09–3.71)	< 0.0001
≥7 (*n* = 921)	9.6	6.97	(5.40–9.00)	< 0.0001

However, when adjusting for key confounders such as labor type, Bishop score, and rupture of membranes in the Cox proportional hazards model, this association was reversed, showing no increased fever risk with more exams. Table 3 presents the final Cox proportional hazard model results. After accounting for confounders, the risk of fever *decreases* as the number of cervical exams *increases*. Specifically, patients with 5–6 exams had a lower risk of fever (HR 0.54) compared to the 1–4 exam group. Patients with ≥7 exams had the lowest risk of fever in the overall cohort (HR 0.24, 95% CI 0.13–0.43, *p* < 0.01) and in the ROM subgroup (HR 0.57, 95% CI 0.30–1.05, *p* = 0.07) compared to those with 1–4 exams.

**TABLE 3:** Overall final Cox proportional hazard model for intrapartum fever.

Number of cervical exams	Hazard ratio [HR] (95% CI)^a^	*p* value
Time from first cervical exam to fever or delivery		
1–4 (*n* = 1473)	Reference	—
5–6 (*n* = 1233)	0.54 (0.31–0.94)	0.03
≥ (*n* = 921)	0.24 (0.13–0.43)	< 0.01
Time from rupture of membranes to fever or delivery		
1–4 (*n* = 1448)	Reference	—
5–6 (*n* = 1200)	1.03 (0.57–1.87)	0.93
≥ (*n* = 912)	0.57 (0.30–1.05)	0.07

^a^ Adjusted for labor induction, Bishop score > 5, ROM on admission, ROM–time interaction.

**FIGURE 1:**
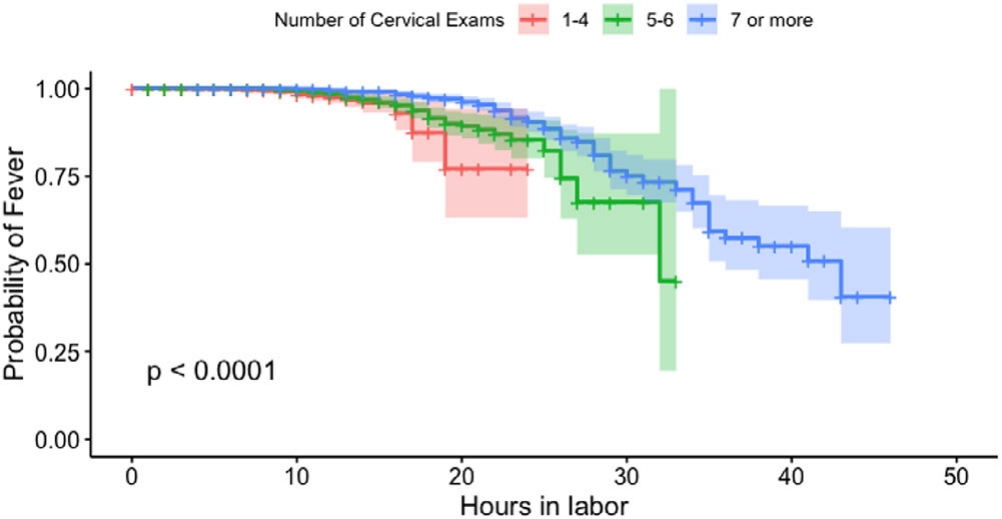
Likelihood of ongoing labor without fever versus hours in labor, full cohort.

**FIGURE A1:**
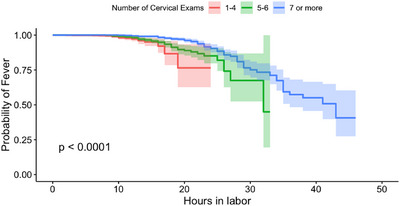
Likelihood of ongoing labor without fever versus hours in labor, nulliparas only.

**FIGURE A2:**
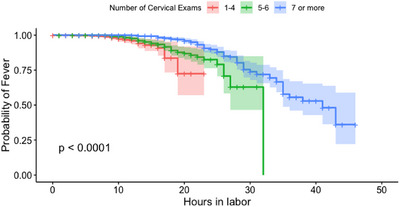
Likelihood of ongoing labor without fever versus hours in labor, cervical dilation < 6 cm on admission.

Figure 1 presents a survival curve showing the likelihood of ongoing labor without fever, stratified by the number of cervical examinations. The reference group (1–4 exams) is depicted by the red curve, while the 5–6 exams and 7 or more exams groups are shown in green and blue, respectively. The curves demonstrate a differential risk of developing fever. The curves illustrate that the reference group has a higher cumulative incidence of fever, as indicated by a steeper decline. Conversely, the 7+ exams group consistently demonstrates the lowest incidence of fever throughout labor. This trend, which is statistically significant (*p* < 0.01), supports the finding that more cervical exams are associated with a reduced risk of fever. Refer to the Appendix (Figures A1, A2) for survival curves corresponding to the subgroup analyses.

Table 4 shows the subgroup analysis for nulliparous patients, which mirrored the findings of the overall cohort. Among nulliparas, those with 5–6 exams had a reduced risk of fever (HR 0.53, 95% CI 0.30–0.95, *p =* 0.032) compared to those with 1–4 exams. The lowest risk of fever was observed in patients with 7 or more exams (HR 0.20, 95% CI 0.11–0.38, *p* < 0.001).

**TABLE 4:** Nulliparas only—Cox proportional hazard model for intrapartum fever.

Number of cervical exams	Hazard ratio [HR] (95% CI)^a^	*p* value
Time from first cervical exam to fever or delivery		
1–4 (*n* = 477)	Reference	—
5–6 (*n* = 608)	0.53 (0.30–0.95)	0.032
≥ 7 (*n* = 601)	0.20 (0.11–0.38)	<0.001
Time from rupture of membranes to fever or delivery		
1–4 (n = 464)	Reference	—
5–6 (*n* = 582)	1.12 (0.59–2.14)	0.724
≥ 7 (*n* = 593)	0.56 (0.28–1.09)	0.086

^a^ Adjusted for labor induction, Bishop score > 5, ROM on admission, ROM–time interaction.

Table 5 presents the subgroup analysis for patients with an initial cervical dilation of less than 6 cm, confirming the same association as the overall cohort. Patients with 7 or more exams had the lowest risk of fever (HR 0.25, 95% CI 0.14–0.46, *p* < 0.001) compared to the reference group.

**TABLE 5:** First cervical exam ≤ 6 cm dilation—Cox Proportional Hazard Model for Intrapartum Fever.

Number of cervical exams	Hazard Ratio [HR] (95% CI)^a^	*p* value
Time From First Cervical Exam to Fever or Delivery		
1–4 (*n* = 1165)	Reference	—
5–6 (*n* = 1169)	0.55 (0.31 ‐ 0.98)	0.043
≥ 7 (*n* = 903)	0.25 (0.14 ‐ 0.46)	<0.001
Time from rupture of membranes to fever or delivery		
1–4 (n = 1142)	Reference	—
5–6 (*n* = 1136)	1.01 (0.53–1.92)	0.974
≥ 7 (*n* = 895)	0.56 (0.29–1.10)	0.092

^a^ Adjusted for labor induction, Bishop score > 5, ROM on admission, ROM‐time interaction.

Table 6 presents the final Cox proportional hazard model for intrapartum fever with an *additional* adjustment for epidural use. The adjustment revealed no significant impact of epidural use on fever risk (*p* = 0.253), although a marginally significant association was observed when evaluating the time from rupture of membranes to delivery (*p* = 0.047). The association between more cervical exams and lower fever risk remained consistent even after adjusting for epidural use.

**TABLE 6:** Overall final Cox proportional hazard model for intrapartum fever—additionally adjusted for epidural use.

Number of cervical exams	Hazard ratio [HR] (95% CI)^a^	*p* value
Time from first cervical exam to fever or delivery		
1–4 (*n* = 1473)	Reference	—
5–6 (*n* = 1233)	0.54 (0.31–0.94)	0.03
≥ 7 (*n* = 921)	0.24 (0.13–0.44)	< 0.01
Time from rupture of membranes to fever or delivery		
1–4 (*n* = 1448)	Reference	—
5–6 (*n* = 1200)	1.04 (0.57–1.89)	0.89
≥ 7 (*n* = 912)	0.59 (0.31–1.09)	0.09

^a^ Adjusted for labor induction, Bishop score > 5, ROM on admission, ROM–time interaction, use of epidural anesthesia.

## 4 DISCUSSION

### 4.1 Principal findings

After adjusting for confounders using time‐to‐event analysis, an increased number of cervical exams was associated with a decreased risk of maternal fever. Although the secondary analysis examining the time from rupture of membranes (ROM) to fever approached statistical significance (*p* = 0.07), the trend remained consistent. This finding was also observed in subgroup analyses of nulliparous patients (*p* < 0.001) and those admitted with cervical dilation < 6 cm. The unadjusted analysis showed a higher fever risk with more cervical exams, likely due to confounding factors such as labor duration, parity, mode of labor, and rupture of membranes. After adjusting for these variables, the Cox proportional hazards model found no independent association between cervical exam frequency and fever risk.

### 4.2 Results in the context of what is known

This retrospective study, conducted in our contemporary cohort, reaffirms the findings of the only other published study, by Cahill et al., that has used a time‐to‐event model to assess the association between the number of cervical examinations and the risk of intrapartum fever in term laboring patients [12]. Both studies found no increased fever risk with more cervical examinations. Notably, our study revealed a negative association. While our analytic approach aligns with the prior work by Cahill et al., our study offers key distinctions: a more contemporary and heterogeneous cohort, subgroup and secondary analyses, and updated contextualization within modern labor management protocols.

Our findings contrast with previous studies that identified cervical exam frequency as an independent risk factor for maternal intrapartum infection. For instance, Gluck et al. (22,183 patients, ≥ 37 weeks) reported that ≥5 vaginal exams were associated with increased febrile morbidity, and Soper et al. found that four or more exams were independently associated with a threefold increase in intra‐amniotic infection risk (OR 3.07, 95% CI 2.53–3.73) [9, 10]. A large single‐center retrospective cohort study by Gomez‐Slagle et al. found that individuals with ≥8 cervical exams had 1.7 times the risk of developing clinical chorioamnionitis compared with those who had 1–3 exams [11].

These studies primarily used logistic regression models that adjusted for labor length and membrane rupture duration as independent variables. However, this approach may not fully capture the complex relationship between cervical exam frequency and labor duration. Logistic regression does not account for the evolving nature of labor, as it treats labor duration as a fixed variable rather than incorporating it as part of a dynamic process over time. The observed association between cervical exam frequency and fever risk may reflect the fact that longer labors, which require more exams, are also associated with higher fever risk. Without adjusting for this time‐dependent relationship, the true impact of cervical exams on fever risk may be obscured.

Time‐to‐event analysis, on the other hand, models the risk of fever as it changes over the course of labor. This approach treats labor duration and cervical exam frequency as time‐dependent variables, which allows for an understanding of how the frequency of exams influences fever risk in real time while adjusting for other time‐varying factors such as membrane rupture. By considering the relationship between these factors as dynamic, time‐to‐event analysis reduces bias introduced by confounding variables and provides a more accurate assessment of their true impact on maternal fever risk.

### 4.3 Clinical implications

Our study findings align with broader research on the implementation and utility of labor induction protocols and more active labor management. Following the increase in elective labor induction at 39 weeks post‐ARRIVE trial, it is important to acknowledge that induction of labor generally involves more frequent cervical exams [15, 16]. While our findings do not directly support a link between cervical exams and improved labor outcomes, they suggest that routinely timed cervical exams as part of a standardized labor management or induction protocol may not contribute to increased febrile morbidity, a concern previously raised in earlier studies. A secondary analysis of a randomized trial comparing time to delivery among labor induction methods by Hamm et al. showed that adherence to cervical exams every 1–2 h in active labor and every 2–4 h in latent labor was associated with lower cesarean rates and reduced neonatal morbidity [17]. Our findings imply that routine and frequent exams, when integrated into a structured labor management protocol, could optimize labor outcomes without exacerbating the risk of febrile morbidity. The inverse association between exam frequency and fever risk may reflect more structured labor management and earlier intervention, particularly in induced or prolonged labors. More frequent exams may serve as a marker of closer monitoring rather than a causal factor.

### 4.4 Research implications

Future research should incorporate time‐to‐event analysis to explore the relationship between cervical exam frequency and fever risk while acknowledging that both time‐dependent models and logistic regression offer valuable insights. While neither method is inherently flawed, they yield different findings due to their distinct handling of confounders, like labor duration and the evolving nature of labor itself. A systematic review of existing studies, alongside prospective research, could help clarify these differences and refine our understanding of how cervical exams affect maternal fever risk. While a randomized trial theoretically could provide higher‐level evidence, ethical and logistical constraints limit feasibility. Time‐to‐event analysis may offer a methodologically robust alternative for evaluating dynamic exposures during labor.

### 4.5 Strengths and limitations

Our study has several strengths, including the use of time‐to‐event analysis to accurately assess the independent effect of cervical exams on maternal fever risk while accounting for labor duration and membrane rupture. This approach enabled a more nuanced understanding of the relationship between cervical exams and fever risk, addressing time‐dependent variables. With a large, diverse sample, we were able to conduct stratified analyses and adjust for clinical confounders, providing insights into specific subgroups, such as those admitted with a cervical dilation less than 6 cm. Although single‐center in design, the inclusion of both academic and community obstetric practice models increases generalizability. Our findings are notably consistent with the prior study by Cahill et al., now offering confirmatory evidence from two large US centers. Additionally, we accounted for epidural use, which, while not a significant factor in our analysis, is important to consider given its established association with intrapartum fever in the literature [18].

However, we acknowledge several limitations. The retrospective design relied solely on documented cervical exams. Despite efforts to ensure accurate recording, it is possible that some exams were performed but not documented, particularly when a junior resident's exam was validated by a senior resident. This potential nondifferential misclassification could introduce some measurement error or information bias; however, the frequency of such misclassification is expected to be minimal and unlikely to differ across exposure groups, thereby limiting its impact on the study's findings. Second‐stage exams, particularly during active pushing, were not included due to inconsistent documentation and limited ability to standardize. This may lead to an underestimation of total cervical exam exposure, potentially introducing nondifferential misclassification.

The study's lower‐than‐expected fever rate suggests potential underpowering, which may affect the robustness of our conclusions. We were unable to adjust for use of antipyretics such as acetaminophen, as indication and timing were inconsistently documented. While this may confound the association between fever and cervical exams, we expect the effect to be nondifferential across exam frequency groups. Lastly, the exclusion of patients who did not reach the second stage of labor, while necessary for consistent risk exposure in the time‐to‐event model, may limit the generalizability of our findings. Future studies might address these limitations and expand on the role of provider type and labor progression in determining the impact of cervical exams on maternal fever risk.

## 5 CONCLUSION

Our large retrospective cohort study of term patients who reached the second stage of labor found an inverse relationship between cervical exam frequency and maternal intrapartum fever risk. Our time‐to‐event analysis allowed us to account for labor duration, providing a more accurate assessment of cervical exam frequency in relation to the time spent in labor. This association remained consistent even with multiple examinations after membrane rupture, and subgroup analyses of nulliparous patients and those admitted with cervical dilation less than 6 cm further supported these findings. Our principal finding—an inverse association between the frequency of cervical exams and fever risk— suggests that increased cervical exam frequency does not inherently increase the risk of intrapartum fever and may be a marker of more active monitoring and labor management. More frequent examinations may be associated with more intensive labor monitoring, earlier detection of complications, and timely interventions.

Previous studies associating increased cervical exam frequency with higher febrile morbidity may have been influenced by the confounding factor of labor duration itself, which is closely linked to both the frequency of exams and fever risk. Future research should explore the relationship between cervical exam frequency and fever risk across diverse clinical settings, with particular attention to labor duration and the timing of exams relative to other interventions.

## CONFLICT OF INTEREST STATEMENT

The authors declare no conflicts of interest.

## Data Availability

The data that support the findings of this study are available from the corresponding author upon reasonable request.
